# Paternal Methionine Supplementation Alters DNA Methylation Patterns in Preimplantation Sheep Embryos

**DOI:** 10.3390/epigenomes10030046

**Published:** 2026-07-06

**Authors:** Jessica Townsend Graybeal, Zeynep Kizilaslan, Mehmet Kizilaslan, Todd Taylor, Hasan Khatib

**Affiliations:** 1 The Department of Animal Sciences and Agricultural Education, California State University, Fresno, CA 93740, USA; jgraybeal@mail.fresnostate.edu; 2 The Department of Animal and Dairy Sciences, The University of Wisconsin, Madison, WI 53706, USA; zkizilaslan@wisc.edu (Z.K.); kizilaslan@wisc.edu (M.K.); toddtaylor@wisc.edu (T.T.)

**Keywords:** DNA methylation, embryos, fetal programming, nutritional epigenetics

## Abstract

**Background/Objectives:** Parental environmental factors can shape developmental outcomes through epigenetic mechanisms that regulate gene expression. While maternal dietary effects on offspring epigenetics have been well characterized, the impact of paternal diet on embryonic DNA methylation remains poorly understood. Here, we investigated the effect of paternal methionine supplementation on DNA methylation patterns in preimplantation embryos in Polypay sheep. **Methods:** Four yearling rams (two control and two methionine-supplemented) were bred to twelve ewes following estrus synchronization and superovulation. Embryos were collected after natural mating and analyzed using whole-genome bisulfite sequencing (WGBS). **Results:** A total of 842 differentially methylated cytosines (DMCs) were identified in embryos derived from methionine-supplemented sires compared to controls, with 835 hypermethylated and 7 hypomethylated. The majority of DMCs were located in intergenic regions, with minimal representation in exonic regions. To assess overlap between parental dietary effects, DMCs identified in this study were compared with those previously reported in embryos derived from methionine-supplemented dams. Eight hypermethylated DMCs were shared between the two datasets, while no hypomethylated DMCs overlapped. To evaluate the functional relevance of differentially methylated genes, we performed siRNA-mediated knockdown of *SSU72*, a gene associated with multiple DMCs. Knockdown of *SSU72* resulted in an average 18% decrease in blastocyst formation rate (*p* < 0.001). **Conclusions:** These results demonstrate that paternal methionine supplementation alters embryonic DNA methylation patterns and that affected genes may play critical roles in early embryonic development, contributing to fetal programming.

## 1. Introduction

Epigenetic regulation during early embryonic development plays a critical role in establishing gene expression patterns that influence long-term developmental outcomes. This concept underlies the Developmental Origins of Health and Disease (DOHaD) hypothesis, which links early environmental exposures to later-life health [1]. Nutritional perturbations, in particular, have been widely shown to influence epigenetic regulation, including DNA methylation, histone modifications, and non-coding RNA expression [2]. Studies involving protein restriction, undernutrition, and high-fat diets have demonstrated that alterations in maternal diet can lead to changes in DNA methylation and gene expression that are associated with long-term metabolic dysfunction in offspring [3,4,5,6]. While these studies establish a clear link between diet and epigenetic regulation, the underlying mechanisms are diverse and not always well defined.

One pathway through which nutrition can directly affect epigenetic regulation is one-carbon metabolism, which regulates the availability of methyl groups for DNA methylation. Methionine, an essential amino acid, plays a central role in this pathway as a precursor to S-adenosylmethionine (SAM), the universal methyl donor [7]. As such, methionine supplementation provides a targeted approach to investigating how methyl donor availability influences DNA methylation dynamics in germ cells and the resulting effects on early embryonic development.

Our previous work has demonstrated that maternal methionine supplementation alters DNA methylation patterns in both oocytes and preimplantation embryos. Notably, oocytes exhibited widespread hypomethylation, whereas embryos displayed a predominance of hypermethylated differentially methylated cytosines (DMCs), suggesting that methyl donor availability may influence the re-establishment of DNA methylation following fertilization. Functional studies further showed that disruption of genes associated with embryonic DMCs impaired blastocyst development, supporting a role for DNA methylation in early embryonic competence [8].

While maternal nutritional effects on offspring development have been extensively studied, the contribution of paternal diet to epigenetic programming remains less well defined. Paternal dietary perturbations such as low-protein intake have been associated with altered metabolic phenotypes in offspring [9], while high-fat diet exposure has been linked to changes in sperm DNA methylation, increased oxidative stress, and impaired metabolic outcomes in subsequent generations [10]. In livestock, we have demonstrated that methionine supplementation in prepubertal rams alters sperm DNA methylation and is associated with phenotypic changes across generations [11,12,13]. Additionally, paternal methionine supplementation has been shown to influence embryonic gene expression, with differences in sperm DNA methylation correlating with transcriptional changes in resulting embryos [14].

Despite these advances, the embryo represents a critical yet understudied stage that links paternal epigenetic alterations to developmental outcomes. While paternal effects on embryo gene expression have been demonstrated, the direct impact of paternal methionine supplementation on embryonic DNA methylation has not been characterized. Therefore, we hypothesized that methionine supplementation in yearling rams would alter DNA methylation patterns in preimplantation embryos.

## 2. Results

### 2.1. Effects of Paternal Methionine Supplementation on DNA Methylation Patterns in Embryos

**Figure 1:**
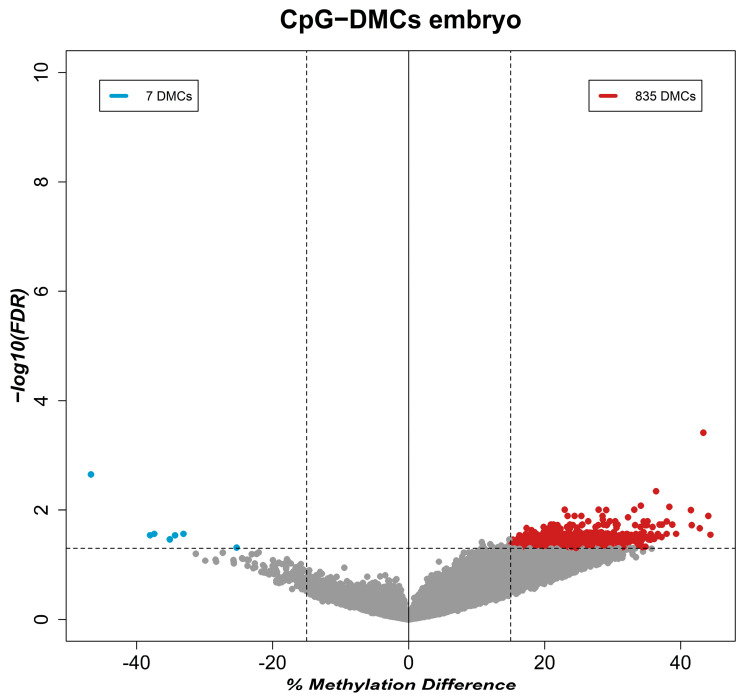
Volcano plot of DMC analysis in the genomic DNA of the embryos. DMCs were defined as those with a methylation difference (x-axis) greater than 15% between treatment and control animals, with a q-value < 0.05 (y-axis) as the threshold. Blue and red dots represent DMCs hypomethylated and hypermethylated in the methionine-treated group compared to the control group, respectively.

To investigate whether methionine supplementation in the paternal diet affects DNA methylation in embryos derived from methionine-treated rams, we performed WGBS on five treatment and seven control pools. No morphological differences were found between the embryo groups. Additionally, no clear pattern of differences or stratification was observed between blastocyst and morula pools, suggesting similar methylation profiles, as evidenced by diagnostic Principal Component Analysis (PCA) plots and correlation heatmaps. A total of 842 DMCs were identified in embryos derived from methionine-supplemented sires (q-value < 0.05; methylation difference > 15%) (Supplementary Table S1). Of these, 835 DMCs were hypermethylated, while only seven DMCs were hypomethylated (Figure 1). Genomic annotation revealed that only two DMCs were located within exonic regions, whereas the remainder were located in intergenic regions. When DMCs were annotated within ±20 Kb of transcription start sites (TSS), four previously uncharacterized genes were identified in proximity to these differentially methylated loci (Supplementary Table S2). Notably, one uncharacterized gene, *LOC114118287*, predicted to encode an RNA polymerase II subunit A C-terminal domain phosphatase SSU72-like protein 3-like (SSU72-like), harbored six hypermethylated DMCs within its intergenic region. Given the known role of *SSU72* in transcriptional regulation and RNA polymerase II recycling, this gene was selected for downstream functional analyses to evaluate its potential contribution to early embryonic development.

### 2.2. Overlap of DMCs Between Paternal and Maternal Methionine Supplementation

To determine whether similar epigenetic signatures were present in embryos derived from paternal versus maternal methionine supplementation, the DMCs identified in this study were compared with those previously reported in embryos from methionine-supplemented dams [8]. Both experiments were conducted in Polypay sheep and utilized the same methionine dose of 3 g per day. Although supplementation duration differed slightly (147 days in the maternal study and 107 days in the present paternal study), the overall experimental designs were highly comparable. Notably, eight hypermethylated DMCs overlapped between the two datasets (Supplementary Table S3). No overlapping hypomethylated DMCs were detected. Of the eight shared DMCs, three were mapped to the initial regions of chromosomes 18, 19, and 26, with no genes in close proximity. The remaining five were found in unmapped contigs of the ARS_UI_Ramb_3.0 genome assembly, leaving their assignment to chromosomes in future assemblies inconclusive. Nevertheless, the shared hypermethylated loci suggest that both paternal and maternal methionine supplementation may converge on a subset of embryonic genomic regions, potentially representing diet-sensitive epigenetic hotspots. However, the limited overlap also indicates that most methylation changes induced by paternal supplementation are distinct from those observed after maternal supplementation.

### 2.3. siRNA Knockdown of SSU72 Affects Embryo Development

**Figure 2:**
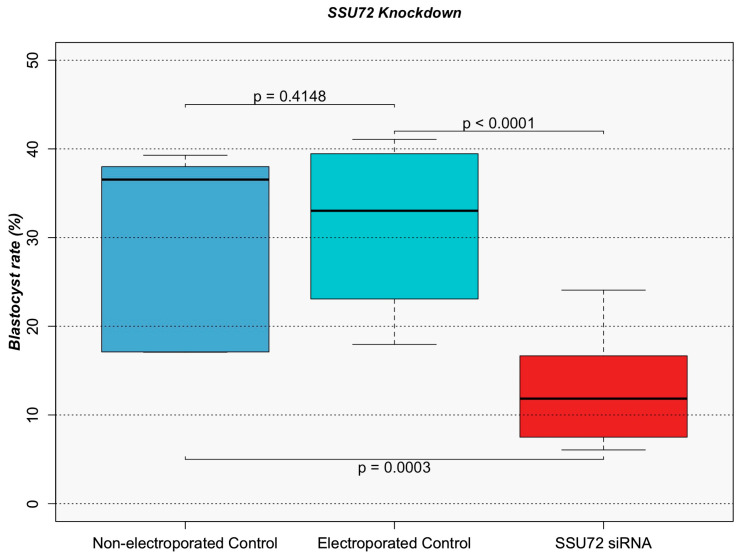
Box plot illustrating the distribution of blastocyst rates in embryos electroporated with *SSU72* siRNA, compared to the electroporated (without siRNA) and non-electroporated controls. Boxes represent the interquartile range, the horizontal line within each box indicates the median, and whiskers denote the minimum and maximum values of the replicates. Statistical significance was assessed using the Wald test, with *p*-values indicated above or below pairwise brackets.

Bovine zygotes were electroporated with siRNA targeting *SSU72* for gene silencing to assess the functional relevance of embryonic DMCs. A total of 247 non-electroporated control embryos, 210 electroporated control embryos (without siRNA), and 233 siRNA-electroporated embryos were used throughout biological replicates. The mean blastocyst rate for five replicates of non-electroporated control is 29.60%, with a maximum of 39.29% and a minimum of 17.07%. Similarly, the mean blastocyst rate for four replicates of the electroporated control is 31.27%, with a maximum of 41.07% and a minimum of 17.95%. In contrast, the mean blastocyst rate for five replicates of siRNA-electroporated is 13.23%, with a maximum of 24.07% and a minimum of 6.06%. Statistical analysis showed no significant difference in blastocyst rates between the non-electroporated control and electroporated control groups (z = −0.815, *p* = 0.4148). In contrast, the blastocyst rate of siRNA-electroporated embryos was significantly lower than that of both the non-electroporated control (z = 3.749, *p* = 0.0003) and the electroporated control groups (z = 4.379, *p* < 0.0001) (Figure 2).

## 3. Discussion

Paternal nutrition plays a crucial role in shaping offspring development through epigenetic mechanisms; however, the direct effects of dietary interventions on embryonic epigenetics remain incompletely understood. Therefore, this study aimed to evaluate DNA methylation in the embryos produced from methionine-treated rams. Notably, we identified 842 DMCs in the embryos of methionine-treated versus control rams, with 835 being hypermethylated. Given that methionine is a key methyl donor in one-carbon metabolism, a diet enriched in methionine would be expected to increase DNA methylation levels in individuals directly exposed. However, previous studies in germ cells have shown the opposite trend, with methionine supplementation often leading to widespread hypomethylation [8,11,14]. Although sperm DNA methylation was not directly assessed in the present study, our previous work has consistently demonstrated that methionine supplementation results in a predominance of hypomethylated DMCs in sperm [11,13,14]. Similar patterns have also been observed in oocytes from methionine-supplemented ewes [8].

In contrast to the hypomethylation previously observed in germ cells, the present study identified a strong bias toward hypermethylation in embryos derived from methionine-supplemented sires. This finding is consistent with our previous observations in embryos derived from methionine-supplemented dams, where a similar predominance of hypermethylated DMCs was detected [8]. The divergence in methylation patterns between germ cells and embryos likely reflects the extensive epigenetic reprogramming that occurs following fertilization. During early embryogenesis, the parental genomes undergo global demethylation, followed by de novo methylation to establish a new epigenetic landscape necessary for development [15]. Although most germ-cell-derived methylation marks are erased during this process, several genomic regions, including imprinted genes [16], transposable elements [17,18], and certain transgenerationally inherited regions [12,13,19] have been shown to escape reprogramming and retain epigenetic information. These retained or preferentially remethylated regions may serve as targets through which parental environmental exposures influence embryonic development. Previous work in sheep has further demonstrated that environmentally induced methylation marks resulting from paternal methionine supplementation can persist across generations [19], suggesting that some of the hypermethylated DMCs identified in the present study may reside within genomic regions that escape post-fertilization epigenetic reprogramming. Together, these findings indicate that the hypermethylated DMCs identified in embryos may not simply reflect direct inheritance of germ-cell methylation patterns. Instead, they likely arise through interactions between inherited epigenetic information and the extensive reprogramming processes that occur during early embryogenesis. Additionally, because methylation was evaluated only in embryos that progressed to the morula or blastocyst stage, we cannot exclude the possibility that developmental selection contributed to the observed methylation patterns, whereby embryos harboring specific methylation profiles may have been more likely to survive to the developmental stages analyzed. Further studies examining methylation dynamics across multiple developmental stages will be necessary to distinguish among these possibilities and determine the functional significance of these loci in fetal programming.

### 3.1. Effects of Paternal Methionine Supplementation on DNA Methylation Patterns in Embryos

To evaluate the effects of paternal diet on embryonic DNA methylation, we performed WGBS on embryos produced from control ewes bred to either control or methionine-supplemented rams. This analysis identified 842 DMCs, of which only two were located within exonic regions, while the remaining 840 were located within intergenic regions. Although intergenic DMCs are often more difficult to interpret than methylation changes within annotated genes, growing evidence indicates that intergenic DNA methylation contributes to the regulation of miRNA expression [20], enhancer activity, and non-coding transcriptional output [21]. In addition, intergenic methylation has been associated with genomic stability and genome organization [22]. Because miRNAs and other non-coding regulatory mechanisms are critical for early embryonic development and embryo-maternal communication [22,23], methylation changes in intergenic regions may influence developmental processes, even when they reside outside protein-coding genes.

Annotation of DMCs within ±20 Kb of TSS identified four previously unannotated genes located near these differentially methylated regions. Two of these loci have predicted functional annotations, while the remaining two (*LOC114117332* and *LOC121816055*) are classified as non-coding RNAs and contain hypermethylated intergenic DMCs. Interestingly, expression data indicate that *LOC121816055* is highly expressed in the descending colon, whereas *LOC114117332* shows elevated expression in the liver, lung, and oviduct, suggesting potential roles in early tissue differentiation. In addition, *LOC121818522* contained two hypermethylated DMCs within an exon and is predicted to encode a basic proline-rich protein 1-like (PRB1-like) protein. Although the functional roles of basic proline-rich proteins are not fully understood [24], related proteins may provide insight into their biological significance. For example, proline-rich acidic protein 1 (PRAP1), is regulated by estrogen, and its overexpression in endometrial cells prior to implantation has been associated with implantation failure [25]. These findings suggest that PRB1-like expression in embryos may play a role in early developmental processes, including implantation, and warrant further investigation.

### 3.2. SSU72 Knockdown and Implications for Early Embryo Development

*LOC114118287*, predicted to encode an RNA polymerase II subunit A C-terminal domain phosphatase SSU72-like protein, harbored six hypermethylated DMCs and was therefore selected as a candidate to evaluate its potential role in early embryonic development. SSU72 is a highly conserved phosphatase that plays a critical role in the transcription cycle through dephosphorylation of the C-terminal domain of RNA polymerase II, thereby regulating processes from transcription initiation to termination and RNA processing [26,27]. In addition to its role in transcriptional regulation, *SSU72* has been implicated in cell cycle progression, including sister chromatid cohesion and chromosome condensation, both of which are essential for proper cell division [28,29]. Disruption of *SSU72* function has also been associated with defects in transcriptional elongation and mitochondrial dysfunction, further highlighting its importance in cellular homeostasis [27,30]. In the present study, siRNA-mediated knockdown of *SSU72* resulted in an approximately 18% reduction in blastocyst formation rates, suggesting that *SSU72* is essential for successful early embryonic development. Reduced *SSU72* expression may disrupt key processes, such as transcriptional regulation and cell division, during early embryonic development, ultimately compromising embryonic viability. The presence of multiple hypermethylated DMCs associated with this gene suggests that altered DNA methylation at this locus may modulate *SSU72* expression and its downstream transcriptional programs. Furthermore, the apparent sensitivity of *SSU72* to nutritional or environmental factors may provide a mechanistic link between paternal diet and embryonic development.

A primary limitation of this study is the low DNA input inherent to preimplantation embryo samples. The limited amount of DNA resulted in low and uneven sequencing coverage. Although approximately 28.7 million CpG sites per sample received some level of coverage, only 82,758 (~0.3%) met the 10× coverage threshold required for reliable methylation calling. Because the likelihood of a CpG site reaching this threshold is influenced by local sequence characteristics, including GC content, mappability, and repeat content, the retained CpGs likely represent a non-random subset enriched for regions that are more readily sequenced. As a result, the findings may not fully reflect genome-wide methylation patterns. The low DNA input also contributed to variation in alignment efficiency and sequencing depth among samples, further reducing the number of CpG sites available for analysis. Consequently, some biologically relevant DMCs may have remained undetected, potentially leading to an underestimation of methylation changes associated with paternal methionine supplementation and of the overlap between these changes and those reported previously in response to maternal methionine supplementation.

Additionally, the use of pooled embryo samples, while necessary to obtain sufficient DNA for sequencing, may have masked variation among individual embryos and limited the detection of treatment effects present only in a subset of embryos. Consequently, the methylation profiles reported here reflect average patterns across embryos within each treatment group rather than embryo-specific responses. Future studies employing advanced low-input sequencing technologies and single-embryo methylation analyses will be essential to achieving a more comprehensive, higher-resolution understanding of DNA methylation dynamics during early embryonic development.

## 4. Materials and Methods

All procedures involving animals were approved by the Institutional Animal Care and Use Committee of the University of Wisconsin-Madison (Protocol ID: A006488).

### 4.1. Supplementation of Rumen-Protected Methionine (RPM) in Diets of Rams

Two groups of yearling Polypay rams were utilized to assess the influence of paternal diet on embryo DNA methylation. A total of four rams were randomly assigned to one of two dietary treatments: a standard basal control diet as described by Gross et al. [11], or the same basal diet supplemented with a top-dress of 3 g rumen-protected methionine (RPM; Smartamine, Adisseo, Alpharetta, GA, USA), corresponding to a 0.22% inclusion rate. This supplementation approximately doubled the basal diet’s methionine content and was considered a nutritional supplement rather than a pharmacological dose. Each ram was individually fed 0.45 kg of their designated diet once daily, beginning at approximately 16.5 months of age and continuing until breeding at approximately 20 months of age, for a total feeding period of 107 days. Between feedings, rams were group-housed and provided ad libitum access to forage, water, and an additional 1 kg of the basal diet.

### 4.2. Superovulation and Synchronization Protocols

To maximize embryo yield, 12 randomly selected Polypay ewes underwent estrus synchronization and superovulation prior to breeding. Briefly, a controlled internal drug release (CIDR) device was inserted vaginally on Day 0. On Day 7, the CIDR was replaced, and 1.0 mL of prostaglandin (EstroPLAN, Parnell, Overland Park, KS, USA) was administered. Beginning on Day 12, ewes received twice-daily injections of Folltropin for four consecutive days. The CIDR was removed on Day 14.5, followed by administration of 1.5 mL PG600 (Merck Animal Health, Rahway, NJ, USA). On Day 16, each ram was randomly introduced to a pen containing three ewes, and marking harnesses were used to confirm successful breeding.

### 4.3. Embryo Flushes

Embryo collections were performed by GenOvations (Lodi, WI, USA) via laparotomy 8 days after rams were introduced to the ewes. Ewes were fasted for 24 h prior to surgery and anesthetized with 2–4 mg/kg propofol, followed by maintenance with isoflurane. Following intubation, ewes were placed in a Trendelenburg position, and a ~1.5 cm abdominal incision was made for laparoscope insertion to visualize the uterus. The incision was then expanded to ~7 cm through the subcutaneous tissue, abdominal musculature, and peritoneum. The uterine horns were exteriorized, and a 20-G Foley catheter was inserted into the distal third of each horn. Each uterine horn was flushed with 50 mL saline, and the effluent was collected through the catheter into a filtered Petri dish. The uterine horns were subsequently returned to the abdominal cavity, and the musculature and skin were sutured [31]. Following the flush, embryos were washed in ABT Complete Flush Media (ABT360, Pullman, WA, USA), evaluated using the International Embryo Transfer Society (IETS) grading system, and placed into 0.5 mL tubes containing 1–2 µL of media for DNA extraction and sequencing. Embryos were pooled to generate five treatment and seven control samples. To reduce genetic variability within pools, embryos from the same donor ewe were pooled whenever possible. While this strategy led to pools containing embryos at different developmental stages, the distribution of stages was similar across treatment groups. All morulae were classified as Stage 4 (compact morulae), and blastocysts ranged from Stages 6 to 8, minimizing the likelihood of systematic bias due to developmental stage differences. Among the treatment groups, four pools contained 6–12 blastocysts, while one pool contained six morula-stage embryos. For the control groups, six pools contained 5–10 blastocysts, and one pool consisted of 11 morula-stage embryos. Pools were constructed to maintain a similar number of embryos per sample while accounting for embryo availability, and all pools underwent downstream analyses.

### 4.4. Whole-Genome DNA Methylation Analysis of Embryos

Embryo samples were processed for whole-genome bisulfite sequencing (WGBS) using the EZ DNA Methylation Direct Kit (Zymo Research, Irvine, CA, USA), which enables bisulfite conversion directly from cellular material without prior DNA isolation. Following bisulfite treatment, libraries were prepared using the Pico Methyl-Seq Library Prep Kit (Zymo Research), which performs whole-genome amplification, adapter ligation, and PCR amplification in a streamlined workflow. Sequencing was performed at the Roy J. Carver Biotechnology Center (University of Illinois at Urbana-Champaign) on the NovaSeq X Plus platform (Illumina, San Diego, CA, USA). Libraries were sequenced on a 25B lane to generate 150 bp paired-end reads. On average, 204M reads were produced for each of the forward and reverse pairs. Base calling and demultiplexing were performed using bcl2fastq v2.20 (Illumina) to generate FASTQ files for downstream analysis.

### 4.5. Bioinformatic Analysis

Paired-end WGBS reads generated from 12 embryo pools (7 control, 5 treatment) were subjected to rigorous quality control and preprocessing. Initial sequence quality assessments were performed using FastQC v0.12.1, followed by adapter and low-quality base/reads trimming using TrimGalore v0.6.1 in conjunction with Cutadapt v4.8 to ensure an ASCII Phred 33 quality score for the obtained reads [32,33,34]. High-quality reads were then aligned to the Ovis aries reference genome (ARS-UI_Ramb_v3.0, NCBI) using Bismark v0.24.2 with Bowtie2, and cytosine-level methylation calls were extracted for CpG sites [35,36]. On average, 33% mapping efficiency was observed for samples, ranging from 26% to 37%.

DMCs within CpG islands were identified using the methylKit v1.32.1 R package [37]. Out of ~28.7 million CpGs per sample, cytosines located on sex chromosomes or mitochondrial DNA were excluded. A conservative coverage threshold of 10× was applied because, given the overall low mapping efficiency, a less stringent threshold would have retained a larger number of CpGs with methylation calls based on very few independent reads, which are highly susceptible to stochastic variation and would compromise the statistical reliability of per-site methylation estimates. Read coverages were normalized across samples using median scaling. Given the inherent challenges of sequencing low-input embryonic samples, our data preprocessing identified 82,758 common CpG cytosines across the samples to be analyzed. Diagnostic analysis of DNA methylation profiles of preimplantation embryo pools was performed through Principal Component Analysis and Pearson’s correlation heatmap. Differential methylation was modeled using a beta-binomial framework, followed by an F-test as described by Feng et al. [38]. Multiple testing correction was performed using the Benjamini–Hochberg false discovery rate (FDR), and cytosines were considered significantly differentially methylated when meeting both q < 0.05 and absolute methylation difference >15% thresholds [39].

Genomic context and functional annotation of significant DMCs were performed using the ARS-UI_Ramb_v3.0 reference genome annotation together with genomation v1.38.0 and GenomicRanges v1.58.0 [37,40]. DMCs were mapped to gene features, including promoters, exons, introns, and intergenic regions, and distances to transcription start sites were calculated to identify associated genes and assess the distribution of methylation alterations across genomic elements.

### 4.6. In Vitro Embryo Production

The *SSU72* gene was selected for siRNA knockdown experiments to determine whether this gene, identified as differentially methylated in sheep embryos, plays a role in the early stages of embryonic development. NCBI BLAST analysis revealed that ovine and bovine *SSU72* share approximately 93% sequence identity. Given this high level of conservation and the close evolutionary relationship between sheep and cattle, we considered the bovine model to provide meaningful functional insight into the potential role of *SSU72* during early sheep embryonic development. In addition, bovine in vitro embryo production systems are well established and offer a practical platform for functional studies due to the greater availability of abattoir-derived ovaries compared with sheep, facilitating the generation of sufficient biological material for experimental validation [41].

Bovine ovaries were obtained from a local abattoir to confirm the cross-species effects. The in vitro embryo production procedure was conducted according to the methods reported Tríbulo et al. [42]. Briefly, cumulus–oocyte complexes (COCs) were collected from ovaries into a sterile beaker containing oocyte collection medium (OCM), and after filtration, washed with OCM. Groups of 50 COCs were transferred to 500 μL of oocyte maturation medium (OMM) covered with 260 μL of light mineral oil (FUJIFILM Irvine Scientific, Santa Ana, CA, USA) for 24 h of oocyte maturation. Following maturation, COCs were washed three times in prewarmed HEPES-TALP and transferred to fertilization dishes containing 1700 μL of IVF-TALP. To be used for fertilization, a straw of frozen–thawed semen was processed with ISolate Sperm Separation Medium (FUJIFILM Irvine Scientific) with a 50% upper layer and a 90% lower layer to obtain a pure and motile sperm sample. The sperm pellet was washed with HEPES-TALP, centrifuged, and resuspended in IVF-TALP. Using a hemocytometer, sperm concentration was measured and diluted to 17 × 10^6^/mL with IVF-TALP, resulting in a final concentration of 1 × 10^6^ spermatozoa/mL in the fertilization dish. Sperm suspension and penicillamine, hypotaurine, and epinephrine (PHE) were added to the fertilization dish to support fertilization. Following 11 h of fertilization, putative zygotes were transferred into a tube, and the cumulus cells were removed from the zygotes by vortexing, washed three times using HEPES-TALP, and placed in groups of 40 in SOF-BE2 culture medium [42], where zygotes were briefly retained before electroporation.

### 4.7. Electroporation with Small Interfering RNAs (siRNAs)

To ensure siRNA delivery before the first cleavage of the embryo, electroporation was performed at the zygote stage, approximately 12 h post-fertilization. For electroporation, zygotes were removed from the culture plate in groups of 40, washed twice with HEPES-TALP, and then washed three times with Opti-MEM (Gibco, Waltham, MA, USA). Each group was then moved through two consecutive 5.7 μL drops containing Opti-MEM supplemented with custom-designed siRNAs (QIAGEN, Germantown, MD, USA) at a final concentration of 1 μM. The siRNA sequences were as follows: 5′-GAAAGGUUCCAGAACUGCATT-3′ and 5′-UGCAGUUCUGGAACCUUUCTG-3′. Based on preliminary tests of different siRNA concentrations, 1 μM siRNA was identified as optimal for delivery and knockdown efficiency. A group of zygotes in the siRNA/Opti-MEM solution was then placed on an electroporation slide equipped with platinum plate electrodes (15 mm in length, 1 mm gap, 1.5 mm in height; Bulldog Bio, Portsmouth, NH, USA). For the electroporated control, a group of zygotes in Opti-MEM alone was placed on an electroporation slide. Electroporation was performed using the following parameters: poring pulses −20 V, 6 pulses, 1.5 ms duration, 50 ms intervals, 10% decay rate, and transfer pulse −3 V/mm, 5 pulses, 50 ms duration, 50 ms intervals, 40% decay rate. Following electroporation, zygotes were washed three times in HEPES-TALP and transferred to a fresh culture plate containing 500 μL of SOF-BEII medium overlaid with 20 μL of light mineral oil (Irvine Scientific). On Day 7.5 post-fertilization, blastocyst formation was assessed to evaluate developmental competence. Blastocyst rates were analyzed in R v4.4.2 using binomial logistic regression to determine the effect of siRNA electroporation. A Wald test was used to assess overall significance across treatment groups, followed by a post hoc multiple testing correction with FDR. Significance was determined at the threshold of *p* < 0.05. 

## 5. Conclusions

This study demonstrates that paternal methionine supplementation alters DNA methylation patterns in preimplantation embryos, highlighting the embryo as a critical stage linking paternal diet to epigenetic programming. Embryos derived from methionine-supplemented sires exhibited a strong bias toward hypermethylation, consistent with our previous findings in embryos from methionine-supplemented dams and suggesting that parental diet may influence the re-establishment of DNA methylation following fertilization. Functional analysis of *SSU72* further demonstrated that genes associated with differentially methylated loci play essential roles in early development, as knockdown significantly reduced blastocyst formation. Collectively, these findings provide new insight into how paternal nutrition influences the embryonic epigenome and underscore the importance of dietary factors in early developmental programming.

## Data Availability

Publicly available datasets were analyzed in this study. These data can be found in the NCBI Gene Expression Omnibus (GEO) repository under accession number GSE333833.

## Supplementary Materials

The following supporting information can be downloaded at: https://www.mdpi.com/article/10.3390/epigenomes10030046/s1, Table S1: All CpGs; Table S2: 20 KB TSS annotation; Table S3: Paternal and maternal overlaps.
